# Substance use is associated with condomless anal intercourse among men who have sex with men in India: a partner-level analysis

**DOI:** 10.1186/s12889-022-13192-y

**Published:** 2022-04-11

**Authors:** Sandeep Prabhu, Shruti H. Mehta, Allison M. McFall, Aylur K. Srikrishnan, Canjeevaram K. Vasudevan, Gregory M. Lucas, David D. Celentano, Sunil S. Solomon

**Affiliations:** 1 Johns Hopkins Bloomberg School of Public Health, Baltimore, MD USA; 2 University of Washington, 1959 NE Pacific St Seattle, Washington, 98195 USA; 3 Y.R. Gaitonde Centre for AIDS Research and Education, Chennai, India; 4 Johns Hopkins University School of Medicine, 1830 E Monument St, Rm 444, Baltimore, MDMaryland 21287 USA

**Keywords:** MSM, India, HIV, AIDS, Condomless anal intercourse

## Abstract

**Background:**

Men who have sex with men (MSM) bear a disproportionately high burden of new HIV infections while lagging behind other populations with respect to engagement across the HIV care continuum. General risk factors for condomless anal intercourse (CAI) among MSM are well studied but there is a paucity of partner-level data, where emerging evidence suggests that much of the variation in condom use occurs.

**Methods:**

MSM were recruited across 10 cities in India using respondent-driven sampling (RDS) from 2016–17. Among the individuals who reported sexual intercourse in the prior 6 months, condom use and partner characteristics of the last 4 partners were captured. Correlates of CAI at the individual and partner level were determined using Poisson regression models using generalized estimating equations and incorporating RDS-II weights, which weights estimates for the participant’s network size.

**Results:**

Among the 8,086 individuals, 21,723 sexual partnerships were analyzed. The prevalence of CAI was 46.9% and most partners were casual or one-time (70.7%) with partner HIV status reported as unknown in 42.6% of the sexual encounters. In multivariable analyses, partner-level characteristics associated with higher likelihood of CAI included unknown partner HIV status (aPR vs. known HIV negative partner: 1.34; 95% confidence interval (CI): 1.27–1.43) and use of alcohol/ drugs prior to intercourse either sometimes (aPR 1.42; 95% CI: 1.33–1.51) or always (aPR 1.31; 95% CI: 1.23–1.41). At an individual level, any HIV treatment literacy was associated with a lower likelihood of CAI (aPR 0.80; 95% CI: 0.74–0.86).

**Conclusions:**

To reduce HIV transmission among this population of MSM across India, combination interventions are likely needed. Interventions targeting substance use and education as well as initiatives to increase self-testing are urgently needed among MSM in India and have the potential to reduce HIV transmission in this high-risk population.

Trial registration

ClinicalTrials.gov Identifier: NCT01686750. Date of Registration: September 18, 2012.

## Background

It is already well documented that men who have sex with men (MSM) lag behind other populations with respect to the HIV care continuum despite bearing a disproportionally high burden of HIV [1–3]. In India, this is compounded by the fact that MSM have a 20-fold higher prevalence of HIV than the general population [4]. MSM in India also have a high burden of alcohol use disorder and substance use, which have been shown to be associated with high risk sexual behavior, including condomless anal intercourse (CAI) [5–7]. Providing accessible and acceptable prevention options aimed at reducing risky sexual behavior remain critical strategies for reducing HIV transmission in this population. The importance of interventions that integrate HIV care with substance use and mental health services has been suggested by prior work, including from our group [5, 8].

In this study, we focused on partner-level risk factors because emerging evidence suggests much of the variation in condom use occurs at a partner-level [9]. Only a few studies have looked at partner-level risk factors among MSM in low and middle-income countries (LMIC) and none have been conducted in India, to our knowledge. Inconsistent condom use during anal intercourse among MSM in India is well documented in the literature but studies on risk factors for condomless anal intercourse (CAI) at a partner-level are not known [1, 2, 10, 11].

Studies looking at partner-level risk factors in other settings have shown that alcohol use prior to intercourse is a risk factor for CAI [12–14]. We seek to identify specific partner-level risk factors as these provide an opportunity to devise interventions to reduce HIV transmission risk [12, 13].

In this sample of 10,024 MSM from 10 Indian cities, we examined individual- and partner-level characteristics associated with CAI.

## Methods

### Study design

Data for these analyses were collected as part of the evaluation assessment of a cluster randomized trial among MSM and people who inject drugs (PWID) in India (ClinicalTrials.gov Identifier: NCT01686750) [15]. The trial was designed to examine the role of an integrated HIV prevention and treatment service delivery model on the uptake of HIV testing across 10 Indian cities. Briefly, 10,024 MSM were recruited in 2016 and 2017 as part of the cross-sectional evaluation assessment of the MSM stratum of this trial using respondent-driven sampling (RDS) that was initiated with 2 “seeds” (individuals identified in the qualitative phase as well-connected in the MSM communities) in each city [15]. The target sample size for each city was 1,000 participants [16].

### Study population

**Fig. 1:**
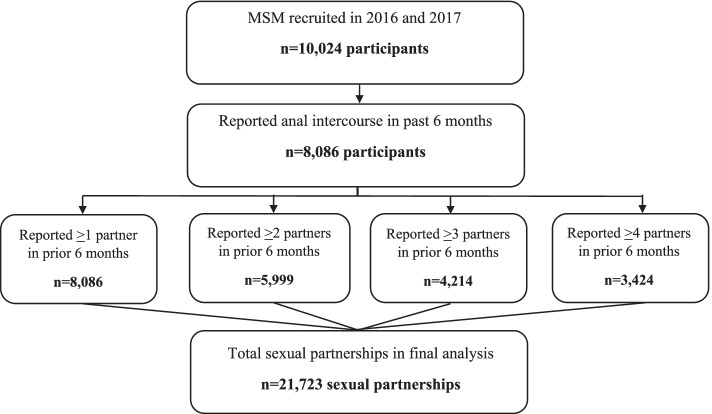
Study flow chart of men who have sex with men (MSM) who reported anal intercourse in past 6 months

Eligibility criteria included: (1) age ≥ 18 years; (2) self-identify as male; (3) report oral or anal sex with another man in the prior 12 months; (4) provide informed consent; and (5) possess a valid RDS referral coupon (except for “seeds”). Participants who self-identified as female or transgender (hijra) were excluded. Of the 10,024 MSM who were recruited, 8,086 reported anal intercourse in the prior 6 months with at least 1 partner and were included in these analyses. Partner characteristics were available for up to the 4 most recent partners per individual. This is outlined in Fig. 1.

### Study procedures

Detailed study procedures have been previously published [3]. After providing verbal consent, participants completed an interviewer-administered electronic survey and provided a blood sample. Rapid HIV testing was performed on-site with appropriate counseling as per Indian Guidelines [17, 18]. Each participant who completed the study was given two coupons to distribute randomly to two other members of his/her peer network (i.e., other MSM in their community whom they knew). Participants were reimbursed Indian Rupees (INR) 250 (USD 4) for completing the study visit which usually lasted about one to two hours. Further, participants could earn an incentive of INR 50 (~ USD 0.80) per eligible participant referred who completed study procedures. All samples were shipped to YR Gaitonde Centre for AIDS Research and Education in Chennai for additional testing.

The electronic survey captured information on socio-demographics, HIV-related risk factors, alcohol use, and substance use. Participants were asked their sexual identities, which are diverse among MSM in India and include *kothis*, who display feminine demeanor and prefer receptive anal intercourse only, *panthis*, who display a masculine demeanor and prefer penetrative anal intercourse only, and *double deckers*, who engage in both receptive and penetrative intercourse. HIV treatment literacy was defined based on several questions about knowledge of HIV treatment. History of sex work was defined by a person reporting their profession to be ‘sex worker’ or said they ever had sex in exchange for money/alcohol/drugs/gifts. Having STI symptoms in prior 6 months was defined based on self-report of unusual anal and/or genital discharge or having pain/ sore in the anal and/or genital region in the preceding 6 months. Anyone who reported unwanted childhood sexual experiences was defined as having experienced childhood sexual abuse. Forced sex was characterized as any encounter where the participant self-reported being forced to have sex when they did not want to. Alcohol use was assessed using the Alcohol Use Disorders Identification Test (AUDIT). Recreational drug use (non-injection and injection) was self-reported using standard measures previously used in India [19, 20]. All participants who reported sex in the prior 6 months were queried in detail up to their four most recent sexual encounters. For each of the four most recent encounters, they were asked questions about the use of alcohol/ recreational drug, gender identity of the participant, type of partner (main vs. casual), frequency of intercourse, type of anal intercourse (receptive vs. insertive) HIV status of the partner and whether they used condoms during the encounter.

All participants underwent on-site rapid HIV testing. HIV RNA was quantified using the RealTime HIV-1 assay (Abbott Laboratories, Abbott Park, Illinois, USA) with a lower limit of detection of 150 copies/ml and HSV-2 status was ascertained an Anti-HSV-2 (gG2) ELISA (IgG) test (Euroimmun Medizinische Labordiagnostika, AG Lubeck, Germany).

### Statistical analyses

Analyses were restricted to individuals who reported anal intercourse in the prior 6 months. Data from RDS “seeds” were excluded in all analyses. The prevalence of CAI (defined as using condoms ‘never’ or ‘sometimes’ when having insertive or receptive anal sex with partner in prior 6 months) was estimated using the RDS-II (Volz-Heckathorn estimator), which weights estimates for network size (i.e., number of MSM in the city whom the participant saw in the prior 30 days). Population summary statistics were estimated with a composite weight, which accounts for the relative population size of adult men 15–59 years of age in each city (assuming a similar proportion of MSM across cities), in addition to the RDS-II weight [21, 22]. Individual and partner level correlates of CAI were explored using Poisson regression models using generalized estimating equations (GEE) to account for multiple partners per individual with associations presented as prevalence ratios (PRs) [23].

Participant and partner characteristics that were associated with a *p*-value < 0.10 were included in the final multivariable model*.* The participant level characteristics included in the final model were age, marital status, education, sexual identity, monthly income, HIV treatment literacy, history of sex work, substance use in prior 6 months (excluding marijuana), STI symptoms in prior 6 months, HSV-2 status, and HIV status. The partner level characteristics that were included in the final model were partner sexual identity, relationship to partner, partner’s HIV status, use of alcohol/ drugs prior to intercourse and frequency of intercourse.

All statistical analyses were performed using Stata version 15.1 software (StataCorp, College Station, Texas).

## Results

### Participant characteristics

Table 1 presents characteristics of study participants and the self-reported characteristics of their last four sexual partners. The median age of participants was 28 years (Interquartile range (IQR): 23–36). About half (47.6%) were never married and 46.9% were currently married or living with a partner of any gender. Over a third self-identified as *panthi* (38.2%) and 22% as *kothi*. Secondary schooling and high school education or more were reported by 42.9% and 40.5%, respectively. Most participants (67.4%) reported an average monthly income > 115 USD.

**Table 1:** Weighted characteristics^a^ by condomless anal intercourse (CAI) among 8,086 men who have sex with men (MSM) and 21,723 MSM sexual encounters in 10 Indian cities

**Characteristic N (%), median (IQR)**	**Condomless anal intercourse (*N*** **= 4,290)**	**Anal intercourse without condoms (*N*** **= 3,796)**	**Total (*N*** **= 8,086)**
** Individual-level characteristics**			
Median age	28 (22, 36)	29 (23, 37)	28 (23, 36)
Marital status			
Never married	2,018 (52.8)	1,577 (42.2)	3,595 (47.6)
Currently married/ living with partner	2,006 (41.2)	2,031 (52.6)	4,037 (46.9)
Divorced/ widowed/ separated/ other	266 (5.9)	187 (5.1)	453 (5.5)
Sexual identity			
Panthi	1,616 (41.1)	1,145 (35.2)	2,761 (38.2)
Kothi	1,007 (18.2)	1,223 (25.9)	2,230 (22.0)
Double-decker	1,210 (27.9)	1,045 (23.8)	2,255 (25.8)
Gay/ MSM	57 (0.1)	80 (2.0)	137 (1.5)
Bisexual	400 (11.9)	303 (13.1)	703 (12.5)
Education			
Primary school or less	894 (18.3)	659 (14.7)	1,553 (16.6)
Secondary school	1,894 (46.1)	1,510 (39.7)	3,404 (42.9)
High school or more	1,502 (35.6)	1,626 (45.6)	3,128 (40.5)
Monthly income			
< $50	526 (14.1)	507 (13.0)	1,033 (13.6)
$50-$115	969 (19.7)	851 (18.3)	1,820 (19.0)
> $115	2,795 (66.2)	2,438 (68.7)	5,233 (67.4)
HIV treatment literacy^b^	1,198 (22.7)	1,702 (35.4)	2,900 (29.1)
History of sex work^c^	1,733 (27.7)	1,192 (22.0)	2,925 (24.8)
Median number of sexual partners in prior 6 months	2 (1, 5)	2 (1, 4)	3 (1, 7)
Childhood sexual abuse^d^	1,075 (20.5)	908 (17.1)	1,983 (18.8)
Forced sex^e^	1,048 (18.7)	856 (17.3)	1,904 (18.0)
Ever sex with woman	2,988 (72.3)	2,502 (73.9)	5,490 (73.1)
Median alcohol use disorders identification test (AUDIT) score	5 (0–11)	1 (0–7)	3 (0–9)
Substance use in prior 6 months^f^ (excluding marijuana)	619 (7.9)	308 (4.9)	927 (6.4)
Substance use in prior 6 months^f^ (including marijuana)	946 (14.9)	532 (9.3)	1,478 (12.1)
Syphilis (self-reported)	163 (2.9)	207 (3.3)	370 (3.1)
STI symptoms in prior 6 months^g^	266 (5.4)	156 (4.2)	422 (4.8)
HSV-2 status			
Negative	3,192 (74.5)	2,517 (69.2)	5,709 (71.9)
Positive	923 (21.6)	1,111 (26.7)	2,034 (24.1)
Indeterminate	169 (3.9)	166 (4.0)	335 (4.0)
HIV status of participant			
HIV-	3,816 (90.7)	2,982 (80.4)	6,798 (85.6)
HIV + , undetectable viral load	193 (4.4)	463 (12.0)	656 (8.1)
HIV + , detectable viral load	281 (4.9)	351 (7.7)	632 (6.3)
**Characteristic N (%), median (IQR)**	**Unprotected anal intercourse (*N*** **= 9,946)**	**Anal intercourse without condoms (***N* **= 11,777)**	**Total (*N*** **= 21,723)**
**Partner-level characteristics**			
Partner’s identity			
Panthi	3,946 (38.9)	3,065 (36.5)	7,011 (37.8)
Kothi	3,998 (29.4)	3,861 (34.9)	7,859 (32.0)
Double-decker	2,677 (20.4)	2,245 (16.9)	4,922 (18.8)
Gay/ MSM	444 (4.7)	372 (6.5)	816 (5.6)
Bisexual	219 (2.3)	319 (4.3)	538 (3.3)
Hijra	493 (4.2)	84 (0.9)	577 (2.6)
Relationship to partner			
Boyfriend	3,101 (20.2)	2,599 (20.9)	5,700 (20.6)
Casual/ one-time partner	7,504 (70.0)	6,586 (71.5)	14,090 (70.7)
Commercial sex worker	1,147 (9.8)	748 (7.6)	1,895 (8.7)
Partner’s HIV status			
Negative	6,450 (47.7)	6,713 (65.7)	13,163 (56.1)
Positive	136 (0.6)	185 (2.1)	321 (1.3)
Unknown	5,191 (51.8)	3,047 (32.2)	8,238 (42.6)
Use of alcohol/ drugs prior to intercourse			
Never	4,914 (51.6)	6,659 (71.5)	11,573 (60.9)
Sometimes	4,163 (28.0)	1,866 (16.0)	6,029 (22.4)
Always	2,697 (20.4)	1,420 (12.4)	4,117 (16.7)
Frequency of anal intercourse with partner			
Less than weekly	8.134 (79.2)	6,857 (78.3)	14,991 (78.8)
At least weekly	3,186 (20.8)	2,847 (21.7)	6,033 (21.2)
Frequency of penetrative anal intercourse with partner			
Never	3,449 (26.0)	3,109 (29.2)	6,558(27.5)
Less than weekly	5,918 (56.5)	4,866 (56.0)	10,784 (56.3)
At least weekly	2,354 (17.5)	1,939 (14.8)	4,293 (16.2)
Frequency of receptive anal intercourse with partner			
Never	4,251 (46.4)	3,182 (40.5)	7,433 (43.6)
Less than weekly	4,890 (43.9)	4,623 (47.1)	9,513 (45.4)
At least weekly	1,838 (9.7)	1898 (12.3)	3,736 (11.0)

^a^Prevalence is RDS-II weighted. Percentages are column percentages. Excluded seeds and individuals who self-identified as hijra

^b^Includes anyone who reports having heard of medicines that control HIV or those who agree that medicines exist which treat HIV/ AIDS

^c^Includes anyone who reports their profession to be ‘sex worker’ or those who stated they have ever had sex for money/ alcohol/ drugs/ goods

^d^Defined as anyone who reports unwanted childhood sexual experiences

^e^Defined as anyone who states that someone has tried to make them have sex when they did not wish to

^f^Includes injection and/ or non-injection use of heroin/ brown sugar, cocaine/ crack, stimulant, buprenorphine, allergy medicine/ antihistamine, painkiller, sedative/ tranquilizer, hallucinogen, inhalant/ solvent, intoxicating tobacco, and other substances in past 6 months

^g^Defined as anyone who reports unusual genital/ anal discharge or pain or sore/ ulcer in the genital/ anal area

### Partner characteristics

8,086 individuals included in the analysis: 3,424 (42.3%) listed 4 partners, 4,214 (52.1%) listed at least 3 partners, and 5,999 (74.2%) listed at least 2 partners. A total of 21,723 sexual partnerships were included. Over a third of partners were *panthi* (37.8%) followed by *kothi* (32%). Most were casual or one-time partners (70.7%), while 20.6% were boyfriends. Participants reported that the HIV status of their partner was negative in 56.1% of partnerships and unknown in 42.6% of partnerships. Drugs/alcohol were used in about 40% of sexual acts with these partners (with similar prevalence regardless of partner HIV status). Frequency of intercourse was less than weekly in most partners (78.8%).

### Prevalence and correlates of CAI

Of the 21,723 sexual partners reported by the 8,086 participants, CAI was reported in 11,777 (46.9%) of the partnerships. In univariable analyses (Table 2), individual-level characteristics associated with increased likelihood of CAI included history of sex work (PR 1.19; 95% confidence interval (CI): 1.11–1.27), having STI symptoms in prior 6 months (PR 1.15; 95% CI: 1.03–1.29), reporting substance use in prior 6 months (PR 1.27; 95% CI: 1.18–1.37), higher median AUDIT score (PR 1.25; 95% CI: 1.22–1.29); partner characteristics associated with higher likelihood of CAI were sexual identity as a double decker (PR 1.07; 95% CI: 1.01–1.15) or hijra (PR 1.5; 95% CI 1.39–1.63), sex work (PR 1.21; 95% CI 1.11–1.32), unknown HIV status (PR 1.42; 95% CI: 1.34–1.5), and use of alcohol/ drugs prior to intercourse (PR 1.5; 95% CI: 1.4–1.61).

**Table 2:** Correlates of CAI^a^ among 21,732 MSM sexual encounters in 10 Indian cities

Characteristic	PR^b^	95% confidence interval	aPR^c^	95% confidence interval
**Individual-level characteristics**				
Age per 10 years	0.98	0.95- 1.02	1.03	0.996–1.07
Marital Status				
Never married	REF		REF	
Currently married/ living with a partner	0.79	0.74–0.84	**0.82**	**0.76–0.88**
Divorced/ widowed/ separated/ other	1.08	0.97–1.21	0.99	0.88–1.11
Education				
Primary school or less	REF		REF	
Secondary school	0.98	0.90–1.06	0.93	0.86–1.01
High school or more	0.82	0.75–0.90	**0.81**	**0.74–0.89**
Sexual identity				
Panthi	REF		REF	
Kothi	0.79	0.73–0.87	**0.88**	**0.78–0.99**
Double-decker	1.01	0.94–1.09	1.02	0.93–1.11
Gay/ MSM	0.63	0.45–0.88	0.76	0.57–1.01
Bisexual	0.89	0.79–1.00	0.94	0.85–1.05
Monthly income				
< $50	REF		REF	
$50- $115	1.11	0.98–1.25	1.00	0.89–1.13
> $115	1.12	1.00–1.24	1.01	0.91–1.22
HIV treatment literacy	0.71	0.66–0.77	**0.80**	**0.74–0.86**
History of sex work	1.19	1.11–1.27	**1.13**	**1.06–1.20**
Number of partners in prior 6 months (per each increase in partner)	1.00	0.996–1.00	**–**	**–**
Childhood sexual abuse	1.06	0.99–1.14	**–**	**–**
Forced sex	1.02	0.95–1.10	**–**	**–**
Ever sex with a woman	0.99	0.92–1.06	**–**	**–**
Median AUDIT score (per 10-point increase)	1.25	1.22–1.29	**–**	**–**
Substance use in prior 6 months (excluding marijuana)	1.27	1.18–1.37	1.06	0.99–1.15
Substance use in prior 6 months (including marijuana)	1.23	1.15–1.32	–	–
Syphilis	0.88	0.72–1.06	**–**	**–**
STI symptoms in prior 6 months	1.15	1.03–1.29	**1.17**	**1.04–1.31**
HSV-2 status				
HSV -	REF		REF	
HSV-1 +	0.83	0.77–0.91	0.95	0.87–1.03
HSV-2 +	1.05	0.91–1.21	1.10	0.95–1.28
HIV status of participant				
HIV-	REF		REF	
HIV + , undetectable viral load	0.59	0.47–0.73	**0.75**	**0.61–0.91**
HIV + , detectable viral load	0.77	0.67–0.90	0.89	0.77–1.04
**Partner-level characteristics**				
Partner Sexual identity				
Panthi	REF		REF	
Kothi	0.87	0.80- 0.93	1.07	0.98–1.16
Double-decker	1.07	1.01–1.15	1.06	0.98–1.15
Gay/ MSM	0.86	0.74–0.99	0.96	0.84–1.10
Bisexual	0.74	0.61–0.90	0.96	0.80–1.13
Hijra	1.50	1.39–1.63	**1.39**	**1.28–1.52**
Relationship to partner				
Boyfriend	REF		REF	
Casual/ one-time partner	1.04	0.98–1.11	0.98	0.92–1.05
Commercial sex worker	1.21	1.11–1.32	0.98	0.89–1.08
Partner’s HIV status				
Negative	REF		REF	
Positive	0.57	0.42- 0.76	0.76	0.56–1.03
Unknown	1.42	1.34- 1.50	**1.34**	**1.27–1.43**
Use of alcohol/ drugs prior to intercourse with partner				
Never	REF		REF	
Sometimes	1.54	1.44–1.64	**1.42**	**1.33–1.51**
Always	1.50	1.40–1.61	**1.31**	**1.23–1.41**
Frequency of intercourse with partner				
Less than weekly	REF		REF	**–**
At least weekly	1.05	1.00–1.11	1.05	1.00–1.11

^a^With RDS-II weights

^b^Prevalence ratio

^c^Adjusted prevalence ratio

In multivariable analyses, individual-level characteristics associated with increased likelihood of CAI included being a history of sex work (aPR 1.13; 95% CI: 1.06–1.20) or reporting STI symptoms in prior 6 months (aPR 1.17; 95% CI: 1.04–1.31); characteristics associated with lower likelihood of CAI were being currently married/ living with a partner (aPR 0.82; 95% CI: 0.76–0.88), at least a high school education (aPR 0.81; 95% CI: 0.74–0.89), *kothi* (aPR 0.88; 95% CI: 0.78–0.99), HIV treatment literacy (aPR 0.80; 95% CI: 0.74–0.86) and being HIV positive with an undetectable viral load (aPR 0.75; 95% CI: 0.61–0.91). (Among those who were married/ living with a partner, those living with male or female partners but not married had lower likelihood of CAI compared to those who were married.)

At the partner-level, in multivariable analysis, CAI was significantly more common if the partner was a hijra (aPR 1.39; 95% CI: 1.28–1.52), had unknown HIV status (aPR 1.34; 95% CI: 1.27–1.43) or drugs/alcohol were used sometimes (aPR 1.42; 95% CI: 1.33–1.51) or always (aPR 1.31; 95% CI: 1.23–1.41) prior to the sexual encounter.

## Discussion

In this large community-based sample of MSM, the prevalence of CAI was nearly half, an alarmingly high number. We present predictors of high-risk sexual behaviors at the partnership level, an area where there has been substantially less investigation compared with traditional overall risk factors for CAI [24, 25]. The importance of such factors comes from increasing evidence that much of variation in condom use occurs at the event-level rather than at the individual level [9, 14, 25]. This study represents one of the largest partner-level risk factor analysis of MSM in the literature and one of the few from a LMIC [11, 12].

We found that the use of alcohol/drugs prior to intercourse increases the likelihood of CAI. Published literature on the relationship between alcohol use and risky sexual behavior among heterosexuals at an event-level has thus far been equivocal, but a positive relationship between the two has been observed among MSM in both high-income settings and LMICs, which we confirm [12, 13, 25–27]. In India, the prevalence of alcohol use in the adult male population is inadequately characterized- available data suggest that high proportions of alcohol users in India meet criteria for hazardous use [28, 29]*.* The easy availability of substances in settings where MSM engage in sex and their role in enhancing the sexual experience contribute to the high rate of alcohol/ drug use, both of which are known risk factors for increased HIV transmission [30, 31]. There are currently millions of opiate and sedative users in India- this problem is likely to worsen in the coming years with the influx of party drugs, such as ecstasy, into the Indian market [32]. Interventions targeting these high levels of substance use including alcohol are needed among MSM in such settings as they have the potential to reduce transmission as demonstrated in these data and improve viral suppression as previously demonstrated [8]. The interventions must be designed with awareness about the current legal environment around these substances.

Unknown partner HIV status (which was the case in 42.6% of sexual encounters in our analysis) was another strong predictor of CAI, relative to HIV-negative partner status. Data from other settings suggests that individuals do not disclose their status to partners due to stigma and rejection, so it is unsurprising that HIV status disclosure to partners is limited [33]. We are also unaware of any data from India about serosorting, whereby an individual engages in CAI with partners who HIV status they are aware of, but not other partners. It is unclear whether this reflects that the communication isn’t happening or that the partner does not actually know his HIV status, but prior studies have estimated that approximately 70% of HIV positive MSM in India are unaware of their status [18]. In fact, the high levels of unknown HIV status among partners (42.6% in our study) suggests that this proportion may even be higher. Tackling these complex challenges in this hidden population in India may require alternative testing approaches such as self-testing and simple education on assessing partner risk. Pre-exposure prophylaxis (PrEP) is another strategy that may help reduce HIV transmission in this population.

The finding of a lower likelihood of CAI among those living with partners but not married compared to those who were married may reflect stable same-sex relationships and requires further work. Future work is needed to develop a conceptual framework of partner-level HIV risks among MSM, similar to those developed for other sexual behaviors [34].

HIV treatment literacy was associated with a lower likelihood of CAI but seen in fewer than a third of individuals in this sample. Tackling this education gap can include strategies to ask about partner status and condom use negotiation strategies. Condom negotiation represents an important skill in reducing risk, but the efficacy of condom use has been shown to be diminished with alcohol use [35]*.* Nevertheless, it remains an important strategy and one that ought to be prioritized in programmatic interventions aimed at HIV risk reduction.

### Strengths and limitations

All data including and partner characteristics were self-reported and subject to recall and social desirability biases. We used trained interviewers who were not from the local community and used validated questionnaires to mitigate these biases. The amount of alcohol use prior to a sexual encounter is not measured and may be important as some studies show a worse outcomes with heavier alcohol use, which we were unable to test but can be explored in future work [26, 36]. The use of alcohol versus drug use prior to intercourse is not measured in the partner and may represent different risks. More nuanced data on drug use, including intended effect of drug use, may provide further insights and understanding into risk behavior. With regards to CAI among those with partners, the breakup of partner by gender is not available, precluding further conclusions from being drawn. Other major strengths of this analysis are the large sample size and a geographically diverse group of participants. We have data on up to four partners per participant, which enabled us to include over 20,000 sexual encounters in the analysis, making this one of the largest partner level analyses of MSM. We used a sampling method (RDS) designed to produce unbiased estimates in the target population. This analysis did not look specifically at condomless receptive intercourse and condomless penetrative intercourse, which we aim to do in future work.

## Conclusions

In this large diverse sample of MSM from India, we demonstrated that unknown HIV status of one’s partner and consumption of drugs/alcohol prior to sexual intercourse were associated with a higher likelihood of CAI. Strategies to improve HIV testing including approaches such as HIV self-testing and interventions to address substance use including alcohol could play an important role in curbing spread of HIV among MSM in India. This study represents one of the largest partner-level analysis of risk factors among MSM in the world and the first from India.

## Data Availability

The data that support the findings of this study are available from the NCA Study Executive Committee, but restrictions apply to the availability of these data, which were used under license for the current study, and so are not publicly available. Data are however available from the authors upon reasonable request and with permission of the NCA Study Executive Committee. The request should include specific aims, hypotheses to be addressed, specific variables that are directly relevant to the proposed analysis, and an explanatory statement on how requested data sets will answer the proposed question(s). Requests should be sent to NCA Study Executive Committee, Department of Epidemiology 615 North Wolfe Street, Baltimore, Maryland, 21,205.

## Abbreviations

MSM: Men who have sex with men
CAI: Condomless anal intercourse
RDS: Respondent-driven sampling
LMIC: Low and middle-income countries
PWID: People who inject drugs
INR: Indian rupees
AUDIT: Alcohol Use Disorders Identification Test
GEE: Generalized estimating equations
PR: Prevalence ratio
QR: Interquartile range
CI: Confidence interval
